# Democracy and Health: reflections and challenges before the 16^th^ Brazilian National Health Conference

**DOI:** 10.11606/s1518-8787.2020054001713

**Published:** 2020-01-23

**Authors:** José Patrício Bispo, Marciglei Brito Morais

**Affiliations:** I Universidade Federal da BahiaInstituto Multidisciplinar em SaúdeVitória da ConquistaBABrasil Universidade Federal da Bahia. Instituto Multidisciplinar em Saúde. Vitória da Conquista, BA, Brasil; II Faculdade PitágorasVitória da ConquistaBABrasil Faculdade Pitágoras. Vitória da Conquista, BA, Brasil

**Keywords:** Health Conferences, Unified Health System, Community Participation, Health Policy, Democracy

## Abstract

This text aimed to analyze characteristics and challenges of the 16^th^ Brazilian National Health Conference based on the conference three thematic axes: Health as a right; Consolidation of the Brazilian Unified Health System (SUS) principles; Adequate and enough funding for SUS. Given the initiatives to dismantle the social security model and the setbacks of social protection policies, to delimitate health in an expanded sense is essential to defend the SUS project. We analyzed the proposal of Universal Health Coverage as an alternative to universal systems. We then presented the restrictions of universal coverage and how the restrictions can threaten the SUS principles. We also discussed insufficient SUS funding and possible worsening in the face of fiscal austerity policies. To strengthen social participation and to monitor the proposals approved at the conference are necessary.

## INTRODUCTION

After 33 years of the anthological 8^th^ Brazilian National Health Conference (CNS), the National Health Council and the sanitary movement sought inspiration precisely at the 1986 conference to structure and organize the 16^th^ CNS. Thus, the 16^th^ conference adopted the theme Democracy and Health: health as a right and consolidation of the Unified Health System (SUS) funding.

The allusion to democracy and its relationship with health are important aspects of the two conferences; however, with very different contexts. The country’s political atmosphere and the social and sanitary movement circumstances are important aspects to reflect on the two periods.

In 1986, Brazil had just ended a 21–year period of civil-military dictatorship, which suppressed civil, political and social rights. In addition to the characteristic barbarities of the violent authoritarian period, the population’s health and living conditions were degrading^1^. With the end of the regime, the Brazilian population began to reject the authoritarian discourse and appeal for democratic values. Feelings that social policies should be inherent to citizenship, organized based on solidarity and redistributive logic, predominated. The sanitary reform movement brought together a myriad of actors and social movements, which gave it great power of social mobilization and political pressure^2^.

In 2019, the scenario is an almost inverted image of the previous period. Democratic values are no longer so appreciated by large portions of Brazilian society. We observe people valuing authoritarianism and the need to restrict rights. This is all enhanced by hate speech, intolerance and defense of what Weffort^3^ called social apartheid. Thus, social policies are increasingly seen as consumer goods. Values of solidarity and social justice are replaced by individualism and meritocracy. Aggravatingly, mobilization of the sanitary community has faded and, consequently, the power to interfere on the direction of politics is different from the past.

Thus, the country’s government context is unfavorable to the SUS consolidation in the face of dismantling the bases of the Welfare State. This moment is an affront to SUS ideas and to more consistent initiatives to destruct it since the proclamation of the Constitution in 1988.

In the face of this complex conjuncture, the themes of the 8^th^ CNS were reread, and the following thematic axes were adopted for the 16^th^ CNS: 1- Health as a right; 2- Consolidation of the SUS principles; 3- Adequate and enough funding for SUS. Thus, this text aimed to analyze characteristics and challenges of the 16^th^ National Health Conference, based on these axes.

### Health as a right

The right to health is not natural, it is not as if the State should always exercise this attribution in any context. Values and culture of a period, as well as the social protection model adopted in the country, will always determine health as a public policy^4^.

Health care models marked by the exclusion of large contingents of the population have once predominated in Brazil^2^. Overcoming these restrictive models, guaranteeing health as a citizenship right, and structuring a public and universal health system are among the most relevant achievements of the Federal Constitution of 1988. Thus, SUS is an important social achievement.

This is not a one-way evolutionary logic. The guarantee of the right to health is not consensual, just as the understanding of the concept of health and how State should act to guarantee it. In contexts of strengthening liberal and individualistic values, social rights are always threatened. Hence the importance of this thematic axis.

More than ever, to reaffirm and to delimitate health in an expanded sense is essential to defend the SUS project. The 8^th^ CNS concept of health is of great relevance for contemporary debate: *Health is the result of conditions of feeding, housing, education, income, environment, work, transportation, employment, leisure, freedom, land access and ownership and health services accessibility*^5^*.* The definition of article 196 of the 1988 Constitution is also important, in which Health is everyone’s right and State’s duty, guaranteed by social and economic policies^6^.

Health is addressed in an expanded perspective and unrestricted to biological aspects in both conceptualities. Thus, the economic and social determinations of health are clear—that is, health is the result of living conditions. Health is only possible with the development of intersectoral policies that promote decent living and working conditions for all the population^7,8^.

The 16^th^ CNS occurs in a context of serious threat to the Brazilian people’s health. Social security is being dismantled and social protection policies, retrogressed. Among the extinct or severely reduced actions, we highlight: housing policy, food security policy, and environmental surveillance actions. In addition, the approvals of the labor reform and of the outsourcing law reform, and the pension reform bill aggravate the precariousness of work and the reduction of the population’s purchasing power.

These measures show the return of the economic growth model of income concentration^9^. This model was experienced in Brazil during the civil-military dictatorship and resulted in the worse living and health conditions of the population^1^. Health preservation in an expanded sense is the greatest challenge for the discussion and for propositions of this thematic axis.

### Consolidation of the SUS principles

The debate on the consolidation of the SUS principles permeates the defense of universal health systems. In addition to the adversities in the national scenario, we should analyze the international circumstance and the strength of multilateral organizations that induce the restriction of health care.

Universal systems began to be questioned under allegations of high costs, low effectiveness and consequent inability of countries to keep them^10^. The new universalism is proposed and understood as the provision of a set of essential services to all citizens^11^, but not the guarantee of all services. It is the offer of a minimum package that limits the right to health and compromises integrality.

The international debate on the conceptions of universality has been accentuated and gained new characteristics in recent years. Criticism of universal systems intensified and the idea of universal health coverage (UHC) was disseminated^12^. Although similar terms, the proposal of universal coverage aligns with the idea of limited package of services and with access by health insurance.

WHO, the main supporter of the proposal, defines UHC in three dimensions: population coverage, service coverage, and financial protection^13^. Law conception is replaced by the financial protection mechanism. It is a restrictive logic, medicalizing, assistentialist and economically motivated. UHC proposal may represent a setback for countries that have health as a right to citizenship and that adopt universal systems^14^.

This debate allies with the conception of Primary Health Care (PHC). Astana Conference in 2018 represented considerable weakening in PHC ideal. While Alma-Ata Declaration evoked for a sense of full and oriented to the principles of social justice PHC, Astana Declaration restricts the sense of PHC to UHC, with reduced state intervention, selectivity, and focus^15^. UHC threatens all the principles of SUS, with restricted universality, abandoned integrality and distorted equity.

Another point worthy of attention in the 16^th^ CNS is the World Bank document that proposes a new SUS reform^16^. This document suggests, among other things, privatization of service provision, competition between providers, limitation of access to specialized services, and sharing of costs. The latter may represent charging services in SUS.

To understand these aspects and how pro-market forces organize and structure themselves inside and outside the country is essential to defend SUS principles. Thus, the 16^th^ CNS is the favorable space to critically analyze these proposals and what they may mean for the future of the system and of the Brazilian population’s rights.

### Adequate and enough funding for SUS

Enough and adequately used financial resources are necessary to guarantee the right to health and to consolidate SUS principles. Funding is a strategic element in the debate of the care models and means carrying the banner of the sanitary movement. Throughout its existence, SUS has never had adequate funding or equivalent to that of countries with universal public systems^2^.

Two indicators may help to understand the SUS underfunding. The first is total health expenditure as a percentage of gross domestic product (GDP). Brazil spends around 8% of GDP on health^17^, while the mean of countries with universal systems is around 12%^18^. The second concerns the percentage of public spending in relation to total health expenditure. A higher proportion of public spending is expected in countries with universal systems. However, Brazil has one of the lowest proportions of public spending (46%), when compared with Latin America (51%), with middle–income countries (55%) and with countries of the Organization for Economic Cooperation and Development (62%)^19^.

This proportion of public investment regards resources directly allocated to SUS. Historically, health policies in Brazil have always stimulated the private sector^2^. Thus, the federal, state and municipal governments spend much more considering the mechanisms of tax waiver, subsidies for civil servants to acquire health insurance, and the existence of private insurances maintained by the governments in parallel with SUS. These mechanisms constitute public funding to the private sector, with total incapability of social participation bodies to control them.

Aggravatingly, fiscal austerity policies influence Brazil to reduce investments in social protection and to reduce the State’s development of public policies^9,19^. Recurrent contingencies on the annual budgets of the ministries of social areas are a proof of this. However, the approval of Constitutional Amendment 95 in 2016 mainly compromises the SUS financial sustainability. With it, health spending has been frozen for 20 years.

SUS progress reached in the last decades will unlikely sustain if the austerity policy maintains^19^. Thus, the realization of the 16^th^ CNS demands the development of a powerful process of social mobilization capable of influencing the reversal of deleterious social, cultural and economic effects of current fiscal and austerity policies.

### Final reflections

The ideological dispute of SUS is at stake. Liberal forces have been able to advance the deconstruction of the values of solidarity and social justice. The idea of searching in the private sector for the care of individuals and families is increasing. In this context, the 16^th^ CNS is a fruitful space to deconstruct liberalizing intentions and to reaffirm and defend SUS as an emancipatory project of society.

Health Conferences and social participation bodies must not adapt to restrictive rules of economic policies. Rejecting a proposal or resolution on the pretext of lack of money is to submit the Sanitary Reform project to the interests of the private sector. The approval of the 16^th^CNS final report is expected, based on the precepts of social security and working as a guiding milestone to expand SUS rather than to restrict it.

To strengthen social participation is important. The conference cannot be only a festive act of momentary mobilization. Monitoring steps need to be valued and converted into instruments of persistent debate and mobilization. Health councils need to revive as enhancing instances of democracy and health.
