# Supplementary material for: Democracy and Health: reflections and challenges before the 16th Brazilian National Health Conference
Source: Rev Saude Publica. 2020 Jan 23;54:16. doi: 10.11606/s1518-8787.2020054001713 (PMC6986867; doi:10.11606/s1518-8787.2020054001713)
Supplement: Translation

# Translation: Democracia e Saúde: reflexões e desafios frente à 16ª Conferência Nacional de Saúde

**Authors:** José Patrício Bispo, Marciglei Brito Morais

**Affiliations:** I Brasil Universidade Federal da Bahia. Instituto Multidisciplinar em Saúde. Vitória da Conquista, BA, Brasil; II Brasil Faculdade Pitágoras. Vitória da Conquista, BA, Brasil

**Keywords:** Conferências de Saúde, Sistema Único de Saúde, Participação da Comunidade, Política de Saúde, Democracia

## Abstract

Este texto teve por objetivo analisar as características e os desafios da 16ª Conferência Nacional de Saúde a partir dos três eixos temáticos da conferência: Saúde como direito; Consolidação dos princípios do Sistema Único de Saúde (SUS); Financiamento adequado e suficiente para o SUS. Diante das iniciativas de desmonte do modelo de seguridade social e dos retrocessos das políticas de proteção social, a delimitação da saúde em sentido ampliado é fundamental para a defesa do projeto do SUS. Analisamos a proposta da Cobertura Universal de Saúde como alternativa aos sistemas universais. Apresentamos as restrições da cobertura universal e as ameaças que podem significar aos princípios do SUS. Discutimos a insuficiência do financiamento do SUS e os possíveis agravamentos diante das políticas de austeridade fiscal. É necessário o fortalecimento da participação social e o monitoramento das propostas aprovadas na conferência.

## INTRODUÇÃO

Passados 33 anos da antológica 8^a^ Conferência Nacional de Saúde (CNS), o Conselho Nacional de Saúde e o movimento sanitário buscaram justamente inspiração na conferência de 1986 para estruturar e organizar a 16ª CNS. Assim, a 16ª conferência adotou o tema Democracia e Saúde: saúde como direito e consolidação do financiamento do Sistema Único de Saúde (SUS).

A alusão à democracia e a relação desta com a saúde são aspectos marcantes das duas conferências, no entanto, com contextos bastante distintos. A atmosfera política do país e as conjunturas social e do movimento sanitário são importantes aspectos para a reflexão sobre os dois períodos.

Em 1986, o Brasil tinha recém encerrado um período de 21 anos de ditadura civil-militar, no qual direitos civis, políticos e sociais foram suprimidos. Além das barbaridades características do violento período autoritário, as condições de saúde e vida da população eram degradantes^1^. Com o fim do regime, o discurso autoritário era fortemente rechaçado pela população brasileira e existia forte apelo aos valores democráticos. Predominavam sentimentos de que as políticas sociais deveriam ser inerentes à condição de cidadania, organizadas com base na solidariedade e na lógica redistributiva. O movimento da reforma sanitária congregava uma miríade de atores e movimentos sociais, o que lhe possibilitava grande poder de mobilização social e pressão política^2^.

Em 2019, o cenário constitui-se em imagem quase invertida do período anterior. Valores democráticos já não são tão apreciados por grandes parcelas da sociedade brasileira. Observam-se posturas de valorização do autoritarismo e da necessidade de restrição aos direitos. Tudo isso potencializado por discursos de ódio, intolerância e defesa do que Weffort^3^ já denominava de *apartheid* social. Assim, as políticas sociais são cada vez mais vistas como bens de consumo. Valores de solidariedade e justiça social perdem espaço para o individualismo e a meritocracia. Como agravante, a mobilização da comunidade sanitária esmaeceu e, consequentemente, o poder de interferir sobre os rumos da política já não é o mesmo de outrora.

Destarte, o contexto governamental do país mostra-se desfavorável à consolidação do SUS frente ao desmonte das bases do Estado de Bem-Estar Social. O momento atual é o de maior afronta ao ideário do SUS e de iniciativas mais consistentes para desestruturá-lo desde a proclamação da Constituição, em 1988.

Frente a essa complexa conjuntura, realizou-se a releitura dos temas da 8ª CNS e foram adotados os seguintes eixos temáticos para a 16ª CNS: 1- Saúde como direito; 2- Consolidação dos princípios do SUS; 3- Financiamento adequado e suficiente para o SUS. Dessa forma, o objetivo do presente texto foi analisar as características e os desafios da 16ª Conferência Nacional de Saúde, a partir dos referidos eixos.

### Saúde como direito

O direito à saúde não é algo natural, como se o Estado devesse sempre exercer essa atribuição em qualquer contexto. A saúde, enquanto política pública, será sempre determinada pelos valores e cultura de um determinado período; consequentemente, pelo modelo de proteção social adotado no país^4^.

Modelos de atenção à saúde marcados pela exclusão de grandes contingentes da população já predominaram no Brasil^2^. A superação desses modelos restritivos, a garantia da saúde como direito de cidadania e a conformação de um sistema público e universal de saúde figuram entre as mais relevantes conquistas alcançadas com a Constituição Federal de 1988. Assim, o SUS constitui-se importante conquista social.

Esta não é uma lógica evolutiva unidirecional. A garantia do direito à saúde não é algo consensual, muito menos é consensual o entendimento do conceito de saúde e qual deve ser a atuação do Estado para garanti-la. Em contextos de fortalecimento de valores liberais e individualistas, os direitos sociais estão sempre ameaçados. Daí a importância do presente eixo temático.

Mais do que nunca, a reafirmação e a delimitação da saúde em sentido ampliado são fundamentais para a defesa do projeto do SUS. O conceito de saúde da 8ª CNS mostra-se oportuno e de grande relevância para o debate contemporâneo: *Saúde é resultante das condições de alimentação, habitação, educação, renda, meio ambiente, trabalho, transporte, emprego, lazer, liberdade, acesso e posse da terra e acesso aos serviços de saúde*^5^*.* Importante também é a definição do artigo 196 da Constituição de 1988 em que Saúde é direito de todos e dever do Estado, garantido por meio de políticas sociais e econômicas^6^.

A saúde é abordada em perspectiva ampliada e não apenas restrita aos aspectos biológicos nas duas conceituações. Evidenciam-se, assim, as determinações econômica e social da saúde – ou seja, saúde é resultante das condições de vida. Não há saúde sem o desenvolvimento de políticas intersetoriais que promovam condições dignas de vida e trabalho para o conjunto da população^7,8^.

A 16ª CNS ocorre num contexto de grave ameaça à saúde do povo brasileiro. Observam-se o desmonte da seguridade social e os retrocessos nas políticas de proteção social. Destacam-se dentre as ações extintas ou fortemente reduzidas: política habitacional, política de segurança alimentar e ações de vigilância ambiental. Somado a isso, as aprovações da reforma trabalhista e da lei das terceirizações, junto com a tramitação da reforma da previdência, agravam a precarização do trabalho e a diminuição do poder aquisitivo da população.

Essas medidas mostram a retomada do modelo de crescimento econômico concentrador de renda^9^. Esse modelo já foi experimentado no Brasil durante a ditadura cívico-militar e resultou no agravamento das condições de vida e de saúde da população^1^. A preservação da saúde em sentido ampliado é o maior desafio para a discussão e proposições desse eixo temático.

### Consolidação dos princípios do SUS

O debate sobre a consolidação dos princípios do SUS perpassa pela defesa dos sistemas universais de saúde. Além das adversidades presentes no cenário nacional, deve-se analisar a conjuntura internacional e a força dos organismos multilaterais que induzem à restrição dos cuidados em saúde.

Os sistemas universais passaram a ser questionados sob a alegação dos elevados custos, baixa efetividade e consequente incapacidade dos países em mantê-los^10^. É proposto o novo universalismo, entendido como a oferta de um conjunto de serviços essenciais a todos os cidadãos^11^, mas não a garantia de todos os serviços. Trata-se da oferta de um pacote mínimo que limita o direito à saúde e compromete a integralidade.

O debate internacional sobre as concepções de universalidade tem se acentuado e ganhado novos contornos nos últimos anos. Intensificaram-se as críticas aos sistemas universais e passou-se a difundir a ideia da cobertura universal de saúde (*universal health coverage *– UHC)^12^. Embora sejam termos parecidos, a proposta da cobertura universal alinha-se à ideia de pacote limitado de serviços e com o acesso por meio de seguros de saúde.

A OMS, principal avalista da proposta, define UHC em três dimensões: cobertura populacional, cobertura de serviços e proteção financeira^13^. A concepção de direito é substituída pelo mecanismo da proteção financeira. Trata-se de uma lógica restritiva, medicalizante, assistencial e com motivação econômica. A proposta de UHC pode representar um retrocesso para países que têm a saúde como direito de cidadania e possuem sistemas universais^14^.

Esse debate está aliado à concepção de Atenção Primária à Saúde (APS). A Conferência de Astana, em 2018, representou considerável enfraquecimento no ideário da APS abrangente. Enquanto Alma-Ata evocava para um sentido de APS integral e orientada aos princípios de justiça social, a declaração de Astana restringe o sentido de APS à UHC, com redução da intervenção estatal, seletividade e focalização^15^. A UHC ameaça todos os princípios do SUS, com a universalidade restrita, a integralidade abandonada e a equidade distorcida.

Outro ponto merecedor de atenção na 16ª CNS é o documento do Banco Mundial que propõe uma nova reforma do SUS^16^. Esse documento sugere, entre outras coisas, privatização da prestação de serviços, concorrência entre os prestadores, limitação do acesso aos serviços especializados e compartilhamento de custos. Esse último pode representar a cobrança de serviços no SUS.

A compreensão desses aspectos e o entendimento de como as forças pró-mercado organizam-se e estruturam-se dentro e fora do país são fundamentais para a defesa dos princípios do SUS. Assim, a 16ª CNS é o espaço propício para análise crítica dessas propostas e o que podem significar para o futuro do sistema e os direitos da população brasileira.

### Financiamento adequado e suficiente para o SUS

São necessários recursos financeiros suficientes e adequadamente utilizados para a garantia do direito à saúde e para a consolidação dos princípios do SUS. O financiamento é elemento estratégico no debate dos modelos de atenção e constitui-se em bandeira histórica do movimento sanitário. Ao longo de sua existência, o SUS nunca contou com financiamento adequado ou equivalente ao de países com sistemas públicos universais^2^.

Dois indicadores podem ajudar no entendimento do subfinanciamento do SUS. O primeiro é o gasto total em saúde em percentual do Produto Interno Bruto (PIB). O Brasil gasta em torno de 8% do PIB em saúde^17^, enquanto a média dos países com sistemas universais gira em torno de 12%^18^. O segundo diz respeito à porcentagem do gasto público em relação ao gasto total em saúde. Espera-se maior proporção de gasto público nos países com sistemas universais. No entanto, o Brasil possui uma das menores proporções de gasto público (46%), quando comparado à América Latina (51%), com países de renda média (55%) e com países da Organização para a Cooperação e Desenvolvimento Econômico (62%)^19^.

Essa proporção de investimento público diz respeito aos recursos destinados diretamente ao SUS. Historicamente, as políticas de saúde no Brasil sempre estimularam o setor privado^2^. Assim, o gasto dos governos federal, estadual e municipal são muito maiores quando se considera os mecanismos de renúncia fiscal, subsídios para servidores públicos adquirirem planos de saúde e a existência de planos privados mantidos pelos próprios governos em paralelo ao SUS. Tais mecanismos constituem-se em financiamento público do setor privado sem qualquer possibilidade de controle pelas instâncias de participação social.

Como agravante, as políticas de austeridade fiscal influenciam o Brasil a diminuir os investimentos na proteção social e a reduzir a atuação do Estado no desenvolvimento das políticas públicas^9,19^. Prova disso são os corriqueiros contingenciamentos dos orçamentos anuais dos ministérios das áreas sociais. Todavia, a principal medida que compromete a sustentabilidade financeira do SUS foi a aprovação da Emenda Constitucional 95, em 2016. Com esta, os gastos em saúde ficam congelados por 20 anos.

É improvável que o progresso alcançado pelo SUS nas últimas décadas seja sustentado caso seja mantida a política de austeridade em curso^19^. Assim, a realização da 16ª CNS demanda o desenvolvimento de um potente processo de mobilização social capaz de influenciar na reversão dos deletérios efeitos sociais, culturais e econômicos das atuais políticas fiscais e de austeridade.

### Reflexões finais

Está em jogo a disputa ideológica do SUS. As forças liberais têm conseguido avançar na desconstrução dos valores da solidariedade e da justiça social. É crescente a disseminação da ideia de se buscar no setor privado o atendimento das necessidades sociais dos indivíduos e famílias. Nesse contexto, a 16ª CNS constitui-se espaço fecundo para a desconstrução dos intentos liberalizantes e a reafirmação e defesa do SUS enquanto projeto emancipador de sociedade.

As Conferências de Saúde e as instâncias de participação social não devem adequar-se aos ditames restritivos das políticas econômicas. Não aprovar uma proposta ou resolução sob o argumento de não haver dinheiro para implementação é submeter o projeto da Reforma Sanitária aos interesses do setor privado. O esperado é a aprovação do relatório final da 16ª CNS fundamentado nos preceitos da seguridade social e que sirva de marco condutor para a ampliação do SUS e não para restringi-lo.

É importante o próprio fortalecimento da participação social. A conferência não pode constituir-se apenas em ato festivo e de mobilização pontual. As etapas de monitoramento precisam ser valorizadas e convertidas em instrumentos de debate e mobilização perene. É necessário reavivar os conselhos de saúde como instâncias potencializadoras da democracia e saúde.
